# Prohibition and the Doctors

**Published:** 1907-08

**Authors:** 


### EDITORIAL NOTES AND COMMENTS.

The Business Office of the JOURNAL-RECORD is 1 1-2 to 5 1-2 South Broad Street.

The Editorial Office is 318-319-320 The Prudential.

Address all Business Commucications to Dr. George Brown, Manager.

Make remittances payable to THE JOURNAL-RECORD OF MEDICINE.

On matters pertaining to the Editorial and Original communications, address Dr. Bernard Wolff, Atlanta.

Reprints of original articles will be furnished at cost price. Requests for the same should always be made on THE MANUSCRIPT.

We will present, postpaid, on request, to each contributor of an original article, twenty (20) marked copies of THE JOURNAL-RECORD OF MEDICINE containing such article.

### PROHIBITION AND THE DOCTORS.

Without attempting any discussion of prohibition as a State measure, there are certain features of it which apply to the medical profession, especially in the restriction of prescribing spirituous liquors for medicinal purposes. Upon these features we feel that some notice is not irrelevant in a journal of the character of the Journal-Record.

The bill requires that the person for whom "pure alcohol" is prescribed shall be personally examined by the physician so prescribing, that the amount of alcohol ordered shall not exceed one pint, that either the doctor or the patient shall procure the alcohol in propria persona, that the prescription shall be filed with the ordinary on the payment of five cents for each prescription, certificate or booze check, whatever the current term for such a document is or shall become,—and some other things which are so strange, new and perplexing that the carnal or rather mortal mind as Mrs. Eddy is wont to term it, cannot adjust itself with sufficient celerity to grasp them all at once.

As law-abiding citizens, is it not pertinent, now that we have a few months before the new order of things becomes effective, to familiarize ourselves with the details so that in our ignorance or unconsciousness we may commit no offense and incur dire punishment.

As a definition is the simplest form of proposition, definitive answers to certain pertinent queries are earnestly desired from anyone who feels qualified to speak from prior experience or superior sagacity and wisdom.

1. What is the definition of pure alcohol?

Does this mean 90 per cent. methylated spirit and is this alone to be prescribed for therapeutic purposes? There is urgent demand for clarity here. We have no desire to convert our patients into human chafing dishes nor to harden their viscera in preservative fluid before death. And we resolved not to do this unless the bill is mandatory and succinctly states that we shall. Then only shall we bow in humble submission and proceed upon our inflammable or sclerotic way.

Pure alcohol as a popular beverage has never been exploited nor as a drug. But perhaps the novel suggestion contained in the bill may possess undreamed-of possibilities in practical application. Pure alcohol cut down so that it would neither stricture nor convert the oesophagus into a cullender with the addition of a little malt or hops extract, ginger ale, coca cola or something else that might offer as a diluent, might be within the pale of the law and at the same time enable the patient to survive the dose.

2. For what diseased conditions, revealed by physical examination, is pure alcohol legally to be prescribed?

This is highly important. It should be so plain that the wayfaring doctor, though a fool, may not err therein. Otherwise confusion worse confounded is sure to result. For instance, that all-gone feeling, that misery in the back, that chilly sensation, that tendency to faintness, of which we hear so much and for which alcohol is alleged to have antidotal properties and remedial virtues of high rank, cannot be revealed by physical examination. Such conditions are subjective, not objective; complaints from the conscious ego, not manifested by visible sign.

To make this matter clear the framers of the bill should have prepared an alphabetical list of diseases and ailments for which pure alcohol may be legally and appropriately prescribed. This list might have been made in two sizes, one large for framing and hanging on the wall of the doctor's office, or small, in the shape of a sticker to paste in his hat or on the fly-leaf of his visiting list, for ready reference, next the obstetrical table and poisons and antidotes.

3. What is the appropriate dose of "pure alcohol," for (a) adult, (b) child?

The several works on materia medica and pharmacology, not foreseeing this action on the part of the Georgia Legislature, have entirely omitted any reference or instruction as to the therapeutic dose and we have no lamp to our feet to point the way to proper prescribing.

It assuredly would not be safe to leave the dose of this vile poison, as it has been graphically termed by some speakers and writers, to a patient's own taste and judgment. No one could conjecture the direful results which might proceed from such indiscretion. By all means the appropriate dose should be carefully designated in accordance with the sufferer's condition, age, sex, color, race or nationality, agreeably to the established facts of toxicology. An overdose would be harmful to the patient, hurtful to the doctor's reputation and distinctly subversive of the morals of the community.

4. What is the proper method of procedure in the instance of the doctor who does his own dispensing; not a druggist-physician, for the law is very clear on this point, but the type of physician who deals out his wares from saddle-bags or other convenient receptacle, the country doctor by preference?

The bill does not permit him to keep pure alcohol in stock and yet accords him the privilege of prescribing it. In such cases how is the doctor to act and how shall the patient procure his "medicine?" Both are placed in embarrassing and hampering positions. The doctor is torn between his imperious desire to aid his patient and his stern and inflexible purpose of abiding by the spirit (not alcoholic), and letter of the law. Is there no way out of this dilemma? Without anticipating those who are to respond to these queries, the writer with due humility and acknowledgment of lack of experience, would make this suggestion: Let the sorely perplexed doctor select a conveniently situated, but perfect neutral hollow tree or log or even a large, sheltering mud-root and therein or thereunder deposit a cache of pure alcohol in honest pint flasks. This hoard may be drawn upon by order or prescription, binding the patient under the most sacred obligation that he will not violate the confidence placed in him by taking more than a pint and that he will duly record the transaction on a memorandum book included in the deposit, nor will he divulge the location of this depot of deposit or bonded warehouse to any one less qualified than himself to know of its situation and purpose. This plan might break off the asperities of the law and satisfy both conscience and physical need.

These are some of the questions which arise and demand answers before the law goes into effect. There may be many other perplexities developing during the course of its operation, but for the present we feel that a proper solution of the difficulties outlined will enable us to sleep with that peaceful serenity which comes from a sense of duty well defined and a crystalization of purpose to carry it out faithfully at the proper time and place.

---

The Atlanta School of Medicine has issued a special edition of its Quarterly Bulletin, illustrating some of the more recent methods of teaching as practiced in the different departments of that institution. The specimen work exhibited in anatomy, physiology, histology, experimental surgery, clinical medicine, gynecology, etc., indicates a well plannd course of instruction and seems to imply that no little individual attention has been given students by the several teachers concerned. The exhibit does credit to the college and its students. It is gratifying to know of such efforts to improve medical education in the South.

It is also announced that arrangements have been perfected to further increase the value of the college hospital as a teaching factor.

---

Dr. Joseph Decatur Bryant, of New York, recently received the degree of Doctor of Laws from the New York University. Dr. Bryant is president of the American Medical Association, having taken office at the recent meeting of that body held at Atlantic City.

